# Hydrocephaly Analysis Supported by Computerized Tomography and Nuclear Magnetic Resonance

**DOI:** 10.1155/2019/5872347

**Published:** 2019-09-30

**Authors:** Tong Zhang, Yawei Zhou, Guohua Su, Dianfeng Shi, Subash C. B. Gopinath, Thangavel Lakshmipriya, Shujing Li

**Affiliations:** ^1^Department of Radiology, Jinan Central Hospital Affiliated to Shandong University, No. 105, Jiefang Road, Lixia District, Jinan, Shandong Province 250013, China; ^2^Department of Hematology, Jinan Central Hospital Affiliated to Shandong University, Jinan, Shandong Province 250013, China; ^3^Equipment Management Office, Jinan Central Hospital Affiliated to Shandong University, Jinan, Shandong Province 250013, China; ^4^Department of Internal Medicine, Jinan Central Hospital Affiliated to Shandong University, Jinan, Shandong Province 250013, China; ^5^School of Bioprocess Engineering, Universiti Malaysia Perlis, 02600 Arau, Perlis, Malaysia; ^6^Institute of Nano Electronic Engineering, Universiti Malaysia Perlis, 01000 Kangar, Perlis, Malaysia

## Abstract

Hydrocephalus is widely known as “hydrocephaly” or “water in the brain,” a building up of abnormal cerebrospinal fluid in the brain ventricles. Due to this abnormality, the size of the head becomes larger and increases the pressure in the skull. This pressure compresses the brain and causes damage to the brain. Identification by imaging techniques on the hydrocephalus is mandatory to treat the disease. Various methods and equipment have been used to image the hydrocephalus. Among them, computerized tomography (CT) scan and nuclear magnetic resonance (NMR) are the most considered methods and gives accurate result of imaging. Apart from imaging, cerebrospinal fluid-based biomarkers are also used to identify the condition of hydrocephalus. This review is discussed on “hydrocephalus” and its imaging captured by CT scan and NMR to support the biomarker analysis.

## 1. Introduction

**Figure 1:**
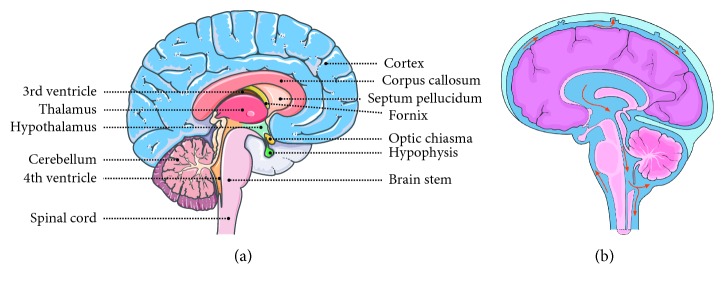
Comparison of normal brain and hydrocephalus brain: (a) normal brain; (b) hydrocephalus brain.

**Figure 2:**
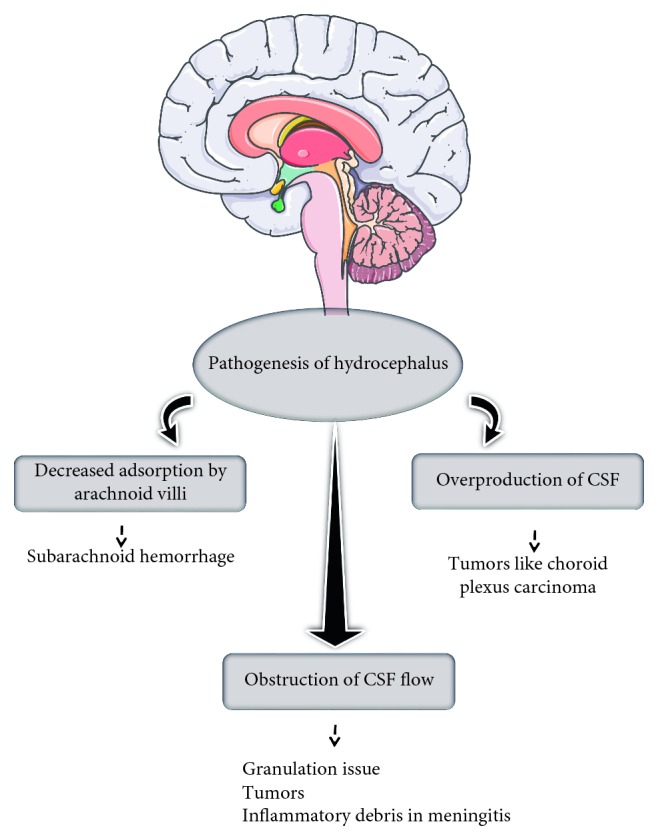
Pathogenesis of hydrocephalus. The ways of formation and details are explained.

**Figure 3:**
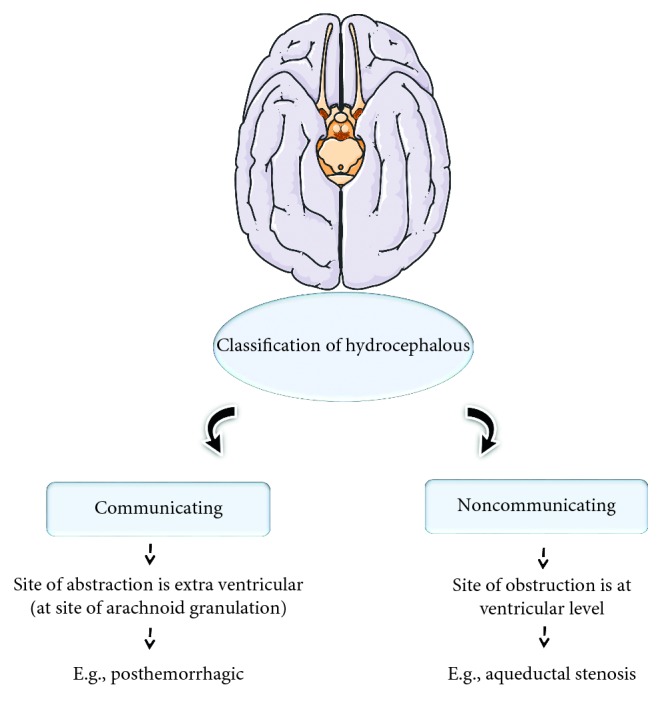
Classification of hydrocephalus. Examples of two types, communicating and noncommunicating, are shown.

Hydrocephalus is the cerebrospinal fluid (CSF) accumulation in the cavities or ventricles of the brain. It causes the enlargement of the skull, which leads to the increment of pressure in the intracranial, and it can be the fatal. In most cases, hydrocephalus is congenital and present during the birth. It also generally happened with children and adult over 60 years; however, some cases with young adults were reported [1, 2]. The comparison of the normal brain (Figure 1(a)) and hydrocephalus brain (Figure 1(b)) shows the obvious differences. Usually under normal condition, CSF flows through the spinal cord and brain, but cases reported with a blockage prevent the normal flow of CSF and decrease the ability of blood vessel to absorb CSF, and in other cases, the excess CSF has been produced by the brain. The above physiological abnormalities cause the increment of CSF, yield pressure to the brain, and damage the brain tissue.  Figure 2 explains the pathogenesis of hydrocephalus that has been shown in the past. With hydrocephalus, “hydrophilic” children face mental and physical disabilities which are the challenges. They have the problems of gait instability, subtle memory, hand and arm dysfunction, and cognitive defeats. Treatment of hydrocephalus at the earlier stages reduce the above disabilities [3]. Before the apparent swelling appearance is being found, hydrocephalus should be diagnosed at very early suspected stage. In general, hydrocephalus is classified into two types, namely, communicating and noncommunicating. The example of communicating is posthaemorrhagic, and aqueductal stenosis is for noncommunicating (Figure 3).

Different approaches have been used to diagnose the hydrocephalus, which include physical, neurological, and imaging tests. In particular, imaging studies show a great accuracy on hydrocephalus. Imaging reflects the enlarged size of the ventricles, caused by CSF. Three main types of imaging tests were popularized to identify the hydrocephalus, which includes ultrasound, CT (computerized tomography or computerized axial tomography), and NMR (nuclear magnetic resonance)/magnetic resonance imaging (MRI). Among the above three methods, MRI shows the detailed image of hydrocephalus under the magnetic field, whereas CT exposes some amount of radiation [4, 5].

## 2. Congenital Hydrocephalus

**Figure 4:**
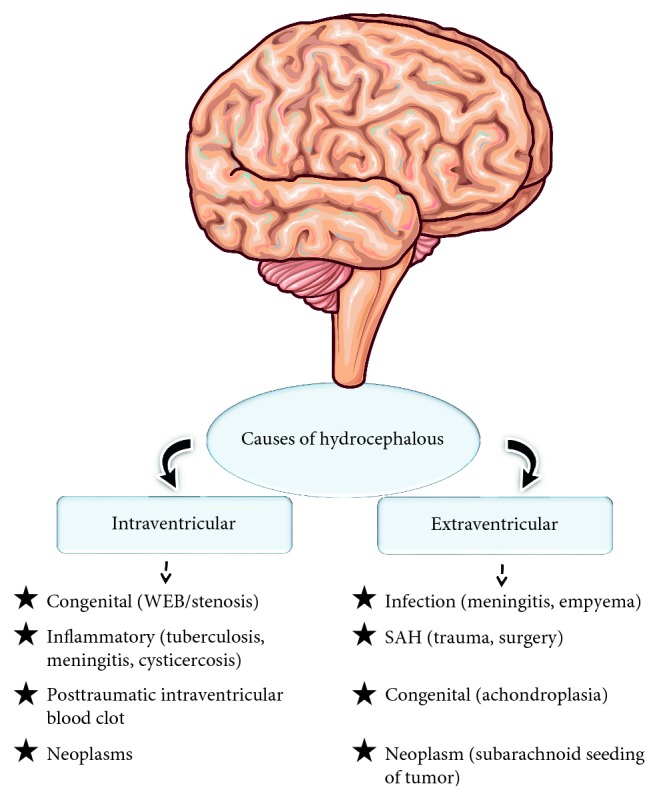
Causes of hydrocephalus. Examples of hydrocephalus caused in extraventricular and intraventricular are shown..

Various reasons have been identified for the cause of hydrocephalus such as tuberculosis, meningitis, congenital, and neoplasm (Figure 4). Among them, the following are considered as the primary reasons for hydrocephalus.

### 2.1. Neural Tube Defects

Neural tube closure (neural tube defeat (NTD)) is a common birth disorder in newborn and the fetus, affecting one in every 1000 births worldwide [6]. NTD is an opening in the brain or spinal cord, which occurs at the earlier stages of human physiological development. The embryo stage of the spinal cord starts as a region of flat and then rolls into a tube after 28 days of pregnancy, called neural tube [7]. NTD develops when the neural tube is not closed completely. Disturbance in the sequential events of embryonic neurulation cause the detects (anencephaly and spins Bifida). There are two types of NTDs such as open and close ones. The common type of NTD is an open type, which occurs when the spinal cord or brain are exposed during the birth through a defect in the skull or vertebrae. Spina bifida, encephalocele, and anencephaly are the examples for open NTDs. Only rare cases are found to have closed NTD, which happens when the skin covers the spinal defeat. The lipomyelomeningocele, tethered cord, and lipomenigocele are the examples for the closed NTDs. Since NTDs are directly linked to the neural plate abnormal closure, it leads to the hydrocephalus [8].

### 2.2. Aqueductal Stenosis

Aqueductal stenosis is one of the major causes of hydrocephalus, particularly congenital hydrocephalus. It is the blockage on the passage of aqueduct of Sylvius, which is the narrow channel (approximately 15 mm) connecting the third ventricle to the fourth ventricle in the dorsal midbrain [9, 10]. This causes the narrowing of aqueduct of sylvius, making CSF fluid not being able to circulate [11, 12]. The aqueductal stenosis is divided into four types, which are stenosis, forking, septum formation, and gliosis. The best diagnosis method of aqueductal is made by MRI [13].

### 2.3. Dandy–Walker Syndrome

Dandy–walker syndrome is a congenital brain malformation involving the fluid-fill around and inside the cerebellum (Figure 1(a); back area in the brain is needed for coordinating the movement) [14, 15]. An enlargement of the small channel called the fourth ventricle causes the Dandy–Walker syndrome. Since this ventricle allows the fluid between the lower and upper parts of the spinal cord and brain and due to the enlargement, there is an absolute lack of area between two cerebellar hemispheres. The formation of cyst occurs near the lower skull, and this size increment leads to hydrocephalus.

### 2.4. Chiari Malformation

**Figure 5:**
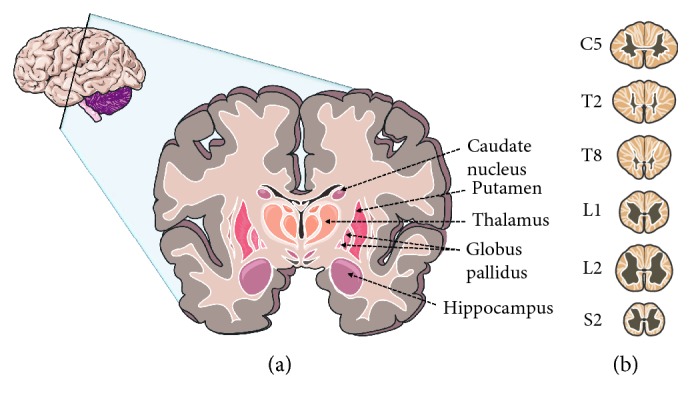
Typical cross sections. (a) Brain; (b) spinal cord.

Chiari malformation is a structural defect in the base of the cerebellum and the skull [16, 17]. It occurs when the part of the skull is abnormally small or misshapens, pressing the brain and cerebellum ultimately forcing it down into the spinal canal and the foramen magnum. It blocks or controls the flow of CSF (cerebrospinal fluid), which circulates nutrients and filters the chemicals from the blood. Chiari malformations are classified into four types, namely, type I, type II, type III, and type IV. Type I forms when the cerebellar tonsils (lower part of the cerebellum) extend into the foramen magnum (opening at the base of the skull). Type II appears usually in the childhood stage. In this case, both the brain tissue and the cerebellum protrude into the foramen magnum. And, also the nerve tissue, which connects two halves of the cerebellum, is found to be missed or only formed partially. Types III and IV occur rarely. In case of type III, there is an involvement of the protrusion herniation with the cerebellum and brain stem via the foramen magnum and also in the spinal cord. Type IV involves the undeveloped or an incomplete cerebellum. It has been found that most of the children born with type II Chiari malformation exhibit hydrocephaly. CT-scan and MRI have been commonly used to diagnose Chiari malformation. These are routinely followed in the diagnosis and follow ups to elucidate the progression of hydrocephalus in patients [18]. To elucidate this, the typical section of the brain (Figure 5(a)) and spinal cord is displayed (Figure 5(b)).

## 3. CT-Scan for Hydrocephaly

**Figure 6:**
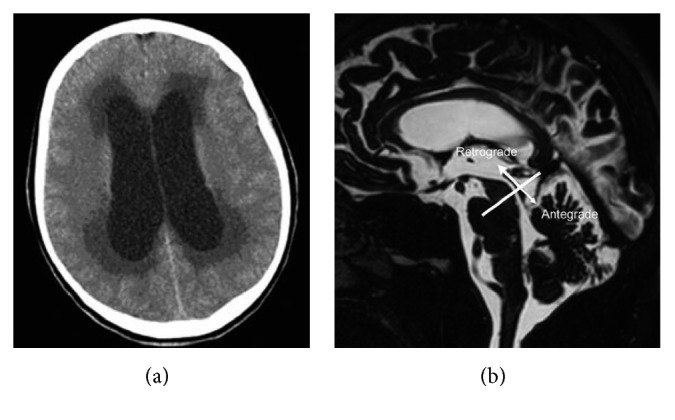
(a) Hydrocephaly image by CT scan (the image is obtained from Fitz and Harwood-Nash (1978)). (b) MRI obtained by steady-state technique (the image is obtained from Ringstad et al. [20]).

Computerized tomography scan (CT or CAT scan) is a diagnostic tool that helps to observe the images of the scanned area by combining the computer technology and a series of X-ray images [19]. CAT scan helps to differentiate the abnormal and normal structures of the organs; the differentiated profiles are used to identify the abnormalities of the infected organs, so that it is considered as an important technique in the field of medical imaging. Since CT scans give more detailed information than X-ray, it has been widely used to diagnose the hydrocephalus. The imaging for the dilation of the lateral ventricle with hydrocephalus is shown in Figures 6(a) and 6(b) [21].

In the CT scan, the prominent temporal horns are the primary indicators among other observations. Usually, with the transverse direction, the third ventricle in diameter of >5 mm has been notified as the abnormality [22]. Balloon appearance with the frontal horn of periventricular hypodensity can be found in obstructive hydrocephalus. Additionally, the ventricular SRC index has also been utilized. The Ventricular SRC Index is estimated by the distance between the anterior tips of frontal horn/bifrontal diameter at the same level (it is from the inner table of the skull). The normal measurement is considered to be thirty percent. Usually, the active hydrocephalus has been marked by the increment in the volume of ventricular. The activity is monitored by the symptoms of the pronounced clinical observation and by the progression follow-up in the CT analysis. The usage of CT scans is prevalent among the hydrocephalus community owing to the utilization of multiple CT scans. Considering the dose exposure of radiation, it has been utilized with a CT scan as 100 to 500 folds more than the routine traditional radiography [23]. This is relatively higher exposure of radiation, whereas MRIs can be preferred in order to eliminate the radiation exposure. However, several patients have no option between MRI and CT scans. It is due to the reason the unavailability of an MRI facility in all the hospitals and in addition to various other medical reasons, the patient does not have an option with MRI. However, the reports have indicated that the utilization of CT scans was enhanced during the last 2 decades and more common with paediatrics. On the other hand, hydrocephalus with children is increasing and considered to be the population that is getting susceptible due to the higher frequency of imaging [24].

## 4. Magnetic Resonance Imaging for Hydrocephalus

As stated above, CT scan uses the X-ray, which has been noticed to cause the side effect due to the exposure to several rounds of scanning [25]. The alternate obvious option is MRI, which uses the powerful magnetic field and pulses of radio frequency for the efficient scanning. The methods of in vivo MRI and spectroscopy were well-set for the past 2 decades. Recent potentials of these methods to analyse the function of the human brain become a fast-growing field of study. NMR commonly shows several lesions and higher participation than CT. The visibility and regions of lesions recognized by NMR are same as those of autopsy obser[[parms resize(1), pos(50, 50), size(200, 200), bgcol(156)]]ed by CT may disappear with repeated examinations, but lesions recognized by MRI will not resolve with the repeated analysis. Different approaches or methods have been developed for the MRI.  Figure 6(b) shows the hydrocephaly image taken by the MRI [20].

MRI works mainly with the hydrogen nucleus, proton in the water, and fat in the tissue [26, 27]. Protons are aligned under a magnetic field; and the radio frequency causes the hydrogen nuclei to resonate. MRI usually scans with 0.5- and 1.5-tesla field strength [25]. MRI observations show convincing results of the morphological appearance and pathological, analytical details of the brain. It helps to distinguish between the normal and pathological brain tissues. In addition, it reveals the dynamics of different neuromuscular diseases. It has been widely agreed that MRI with hydrocephalus is a concrete approach for the analysis of the pathophysiology and pathogenesis, particularly with the periventricular tissue.

## 5. Computerized Tomography versus Magnetic Resonance Imaging

As stated above, the basic difference between CT-scan and MRI is that X-ray has been used in the former case, whereas magnetic field and radio frequency are used in the latter case. Even though different advantages were proposed for both CT and MRI, there is a clear-cut difference between these two in terms of potential merits. Clear images of tissue or bone and infrastructures of an internal organ can be obtained by MRI and considered to be better than CT-scanned images. Contrast dye can be incorporated into the MRI specimen, making a clear distinction for the cancer tissue [27]. However, the analysis with MRI is noisy and consumes a longer time.

## 6. Other Imaging Systems for Hydrocephaly

Apart from CT and NRI, other imaging systems are taken into the consideration for imaging hydrocephaly. 3D ultrasound was used to detect the X-linked hydrocephaly [28]. Not only that, the cytomegalovirus infected pregnant women were affected by fetal hydrocephaly and hydrocephalus syndrome, which were effectively imaged by the ultrasound [29]. Transvaginal sonographic detection was utilized to identify hydrocephaly during the postmenstrual weeks of 15 and 20; this helps to diagnose hydrocephaly before the gestational age [30]. Moreover, machine learning methods were used to detect hydrocephaly efficiently, and hydrocephalus was disproportionately identified by the automated classification model [31].

## 7. Cerebrospinal Fluid Biomarkers and Detection of Hydrocephalus

Apart from CT and NMR, cerebrospinal fluid (CSF) biomarkers are also effectively used to diagnose hydrocephalus. Moreover, identification of these biomarkers gives additional support along with CT and NMR. Cerebrospinal fluid is a regulated fluid with molecular diversities on the local neuro-pathophysiological and neurophysiological processes. Therefore, biomarkers from CSF are highly relevant to different neurological related diseases, which include amyotrophic lateral sclerosis, Alzheimer disease, Parkinson disease, and hydrocephaly [32–34]. The elevated CSF protein was found in the aqueductal obstruction hydrocephaly. Increasing level of CSF protein causes the increment in the CSF-parenchymal oncotic/osmatic gradient; ultimately, the volume of CSF increases, contributing to the hydrocephaly. In particular proteins, APP (amyloid precursor protein), L1CAM (L1neural cell adhesion molecule), and NCAM-1 (neural cell adhesion molecule) were found as the efficient candidates for CSF biomarkers. In addition, it has been found that the level of aminoterminal propeptide and TGF-*β*1 is highly related with the congenital hydrocephalus. In other cases, GEAP protein (glial fibrillary acidic protein), vimentin, MBP (myelin basic protein), and CNPase are playing a potential role in developing hydrocephaly.

**Figure 7:**
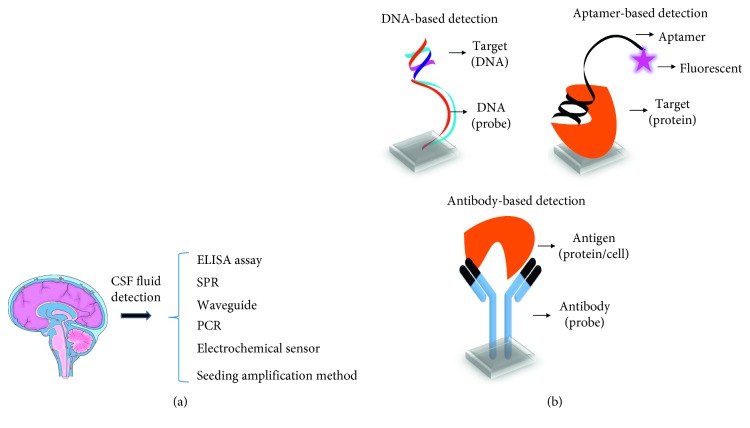
Hydrocephaly detection using CSF fluid biomarkers and identification by a biosensor. (a) Biomarker identification by a different type of biosensor; (b) CSF biomarker detection by different probes such as DNA, antibody, and aptamer.

Identifying these biomarkers using a biosensor gives supporting information for hydrocephaly diagnosis with CT and MRI. Various sensing methods and probes have been used to identify these biomarkers from the CSF. Enzyme-linked immunosorbent assay, polymerase chain reaction (PCR), surface plasmon resonance, waveguide mode sensor, and electrochemical sensor are the predominant sensors to quantify the level of CSF protein (Figure 7(a)). In general, antibody, DNA, RNA, and aptamers were used as the probe to identify and quantify the level of CSF proteins (Figure 7(b)). The level of *β*-amyloid peptide (A*β*) protein from CSF was quantified by surface-enhanced RAMAN spectroscopy [35]. In addition, hydrogel-based biosensor was used to identify the level of CSF amyloid-beta oligomers [36]. The interdigitated electrode sensor was used to identify the CSF protein amyloid-*β* [37]. These types of biosensors with imaging techniques (CT and NMR) give confidence on identification of hydrocephaly and help to treat the people at earlier stages for further medication.

## 8. Conclusion

In the current overview, the basics of hydrocephaly and its analysis by imaging techniques such as computerized tomography and nuclear magnetic resonance are discussed. Both computerized tomography and nuclear magnetic resonance are widely accepted methods and currently in use, owing to their advantages over ultrasound methods. The major advantage of these methods compared to the ultrasound is the ability to penetrate the bone specimen, which cannot be possible by the ultrasound. Between computerized tomography and nuclear magnetic resonance, the latter one is able to differentiate between benign and malignant tissues and the apparent results can be attained. Further, it has been suggested widely that stroke can be recognized by diffused nuclear magnetic resonance as it carries more advantages.
